# Housing Insecurity and Climate-Related Disasters in Underserved Communities: A Call for Action

**DOI:** 10.1093/geroni/igaf122.1432

**Published:** 2025-12-31

**Authors:** Carlos Fuentes, Quenette Walton, Brenda Tijerina, Lauren Gilbert, Omolola Adepoju

**Affiliations:** Tilman J. Fertitta Family College of Medicine, University of Houston, Houston, Texas, United States; University of Houston, Houston, Texas, United States; West Street Recovery, Houston, Texas, United States; University of Houston College of Medicine, Houston, Texas, United States; University of Houston College of Medicine, Houston, Texas, United States

## Abstract

Although natural disasters are equal opportunity events, research suggests that they place a disproportionate burden on minoritized communities. Examining the effects of these disasters is critical to increasing preparedness among our most vulnerable. This study aims to explore community experiences related to housing following natural disasters. Participants included members from three historically underserved Houston communities with Social Vulnerability Index rankings in the 80th percentile. Three town hall-style conversations were held within each community. Participants engaged in facilitated round-table discussions with open-ended questions. Notes were captured in real-time by both an assigned note-taker and a professional graphic recorder. Reflexive thematic analysis was used to identify themes, supported by research triangulation, reflexivity, and member checking for trustworthiness. Analysis identified seven key themes:1) Successive Disasters Exacerbate Problems Driven by Gentrification, 2) Inequitable Distribution of Resources Post-Disaster Results in Reliance on Community-Led Support, 3) Systemic Delays in Relief Services for Underserved Communities must be Addressed by Government Officials, 4) Growing Distrust in Local Government to Address Evolving Post-Disaster Needs, 5) Navigating Complex Insurance Policies While Being Drained by a Disaster, 6) Trickle-Down Unpreparedness Starts at a City Level, and 7) Steps to Prepare for Future Disasters. Systemic inequities in disaster preparedness and response affecting low-income Black and Hispanic communities are evident. Addressing these disparities requires prioritizing equitable resource distribution, infrastructure investments, and community-driven planning and building resilience. Implementing transparent, inclusive strategies to reduce vulnerability and ensure these communities are better supported before, during, and after disasters is crucial.

